# Subclinical Lipopolysaccharide from *Salmonella* Enteritidis Induces Dysregulation of Bioactive Substances from Selected Brain Sections and Glands of Neuroendocrine Axes

**DOI:** 10.3390/toxins11020091

**Published:** 2019-02-02

**Authors:** Anita Mikołajczyk, Dagmara Złotkowska

**Affiliations:** 1 Department of Public Health, Faculty of Health Sciences, Collegium Medicum, University of Warmia and Mazury in Olsztyn, 10-082 Olsztyn, Poland; 2 Department of Food Immunology and Microbiology, Institute of Animal Reproduction and Food Research, Polish Academy of Sciences in Olsztyn, 10-748 Olsztyn, Poland; d.zlotkowska@pan.olsztyn.pl

**Keywords:** LPS from *Salmonella* Enteritidis, brain peptides, HPA axis, HPO axis, HPT axis

## Abstract

Bacterial lipopolysaccharide (LPS) can contribute to the pathogenesis and the clinical symptoms of many diseases such as cancer, mental disorders, neurodegenerative as well as metabolic diseases. The asymptomatic carrier state of *Salmonella* spp. is a very important public health problem. A subclinical single dose of LPS obtained from *S.* Enteritidis (5 μg/kg, i.v.) was administered to discern the consequences of changes of various brain peptides such as corticotropin-releasing hormone (CRH), gonadotropin-releasing hormone (GnRH), thyrotropin-releasing hormone (TRH), galanin (GAL), neuropeptide Y (NPY), somatostatin (SOM), substance P (SP), and vasoactive intestinal polypeptide (VIP) in selected clinically important brain sections and endocrine glands of the hypothalamic-pituitary-adrenal (HPA), -thyroid (HPT), -ovarian (HPO) axes. The study was conducted on ten immature crossbred female pigs. The brain peptides were extracted from the hypothalamus (medial basal hypothalamus, preoptic area, lateral hypothalamic area, mammillary bodies, and the stalk median eminence), and pituitary gland (adenohypophysis and neurohypophysis) sections and from the ovaries and adrenal and thyroid glands. There was no difference in health status between LPS and the control groups during the period of the experiment. Nevertheless, even a low single dose of LPS from *S.* Enteritidis that did not result in any clinical symptoms of disease induced dysregulation of various brain peptides, such as CRH, GnRH, TRH, GAL, NPY, SOM, SP, and VIP in selected brain sections of hypothalamus, pituitary gland and in the endocrine glands of the HPA, HPO, and HPT axes. In conclusion, the obtained results clearly show that subclinical LPS from *S.* Enteritidis can affect the brain chemistry structure and dysregulate bioactive substance from selected brain sections and glands of the neuroendocrine axes. The exact mechanisms by which LPS can influence major neuroendocrine axes are not fully understood and require further studies.

## 1. Introduction

Despite huge progress in medical science over the last years, many chronic diseases such as cancer, mental disorders, neurodegenerative as well as metabolic diseases impose a critical and significant burden on public health. Defining the factors strongly associated with these diseases is of great importance because it may significantly contribute to a decrease in their morbidity. Apart from environmental and genetic factors, the role of infectious agents has been increasingly emphasized. Infectious factors, with viruses being the most common underlying cause, have been estimated to be implicated in up to 18% to 50% of cancers [1,2]. Although several viruses produce disease by promoting malignant transformation of host cells, in other cases mechanisms of malignancy triggered by viral infection are less clear [3,4,5]. Microbes and inflammatory factors may have a role in the development and progression of cancer, responsiveness to particular cancer therapeutics and also in cancer-associated complications [6]. Recently, investigators have observed relationships between the diversity and composition of microbiome and the efficacy of PD-1-based immunotherapy [7,8,9]. By far, the most extensively studied microorganisms in effective tumor therapy by genetic engineering and molecular microbiology are *Salmonella* species with its endotoxins–lipopolysaccharides (LPSs). The mechanisms of *Salmonella* spp. and its LPSs activity in tumor therapy are still being elucidated [10,11,12].

Moreover, it is known that *Salmonella* (Gram-negative facultative anaerobic bacteria) is medically a very dangerous pathogen for humans. Very serious epidemiological problems associated with the introduction of the pathogenic bacteria into the environment and the food chain involve asymptomatic *Salmonella* infection and latent carriers [13]. Although a persistent infection with the same strain of *Salmonella* spp. can last for months or even years without any symptoms of the disease, the prevalence of long-term non-typhoidal serovar carriers in the human population is still not well-known [14]. Diagnosis and identification of carriers are difficult and asymptomatic *Salmonella* infections in both humans and food-producing animals create serious public health threats. Despite numerous studies on asymptomatic *Salmonella* infections and the search for methods to eliminate this pathogen from the food production chain, the problems of *Salmonella* carrier state are still unsolved [15,16,17,18]. *Salmonella* infection in the chronic carrier state is a risk factor for gallbladder cancer. *Salmonella enterica* can promote neoplastic transformations of genetically predisposed cells in the gallbladder [19]. It is important to resolve problems of the carrier state, not only for controlling or eradication but also in relation to aspects of the prediction and prevention of various diseases connected with lipopolysaccharide (LPS) from Gram-negative bacteria. 

LPS is a compound of the cell wall of all Gram-negative bacteria that live in coexistence with humans or are pathogenic for people. LPSs are released from bacteria cells when the bacteria multiply, die or lyse [20,21]. LPS comprises three parts: lipid A, the core oligosaccharide and the O polysaccharide (O antigen). A wide variability in LPS of gram-negative bacteria has been demonstrated [20,22,23,24], and is present not only in the O antigen but also in lipid A. LPS of distinct gram-negative bacteria is involved in the various pathological processes, for example unlike LPS from *E. coli*, LPS from Bacteroides does not decrease the incidence of autoimmune diabetes in non-obese diabetic mice [24]. The variability of intra-species and inter-species LPS hinders the immune response and thus control of bacterial infections and influences different LPS biological activities [25,26,27]. Even LPS from various *Salmonella* serotypes has a varied influence on the immunoreactivity to neuropeptides in vitro [28]. Recent studies suggest that the presence of bacterial LPS may be associated with a range of chronic diseases, including colorectal adenomas and cancer in humans [29,30]. LPSs are well-known as being highly inflammogenic and are capable of causing lethal shock [31]. In addition, many studies of animal models have demonstrated that neurodegeneration involved in Parkinson’s disease (PD) can be induced by LPS inflammation [32,33,34]. LPS inflammation can also play a significant role in the pathogenesis of amyotrophic lateral sclerosis and psychiatric disorders [35,36,37]. The systemic inflammation generated by LPS in a mouse model induces neuroinflammation, amyloidogenesis and memory impairment, which could contribute to Alzheimer’s disease (AD) pathology [38,39].

There is recent evidence suggesting LPS involvement in some neurodegenerative and metabolic diseases, not only in rodents but also in humans. Recent studies have indicated that LPS may be capable of crossing physiological barriers to access the brain and achieve greater levels in the brains of AD patients compared to a control group [40]. Other data show that LPS is associated with metabolic disease in humans. A high serum LPS activity is strongly associated with cardiometabolic disorders [41]. Most of the studies observed higher serum concentrations of LPS or lipopolysaccharide-binding protein in diabetic patients than in healthy people [42]. LPS levels were highest in the obese-diabetic group [43]. The increase in LPS serum concentration was related to a smaller amount of fecal *E. coli*, increased triglyceride levels, waist circumference and central adiposity in obese female youths [44]. The limitations of these studies were a lack of research techniques that target kinds of LPS from specific bacterial groups and some problems with identification of LPS markers for endotoxins, which may remain in various tissues and organs for a long time [45]. There is a well-known case of a laboratory worker with polyneuropathy, encephalopathy, and parkinsonism following an accidental exposure to LPS from *Salmonella* Minnesota, whose organism could not detoxify LPS even 14 years after exposure to a high dose of *S*. Minnesota LPS [46].

Taking into consideration all the above-mentioned, this experiment was conducted using a low single dose of LPS obtained from *S.* Enteritidis that does not lead to any clinical symptoms of disease (subclinical LPS), which can hypothetically occur during the asymptomatic carrier state of *Salmonella* spp. To our knowledge, this is the first report of investigating the influence of a single subclinical dose of LPS obtained from *S.* Enteritidis on selected brain peptides of the hypothalamic-pituitary-adrenal (HPA), -thyroid (HPT), -ovarian (HPO) axes, with the pig used as a biomedical model. Since the study of human hypothalamic pituitary path regulation is impeded by inaccessibility of tissue for biopsy, animal experiments are still essential and appropriate animal models are irreplaceable. Since pigs are phylogenetically closer connected to humans than rodents and the similarities between humans and pigs in terms of physiological, biochemical, and immunological properties are relatively well-known, the domestic pig was chosen as an experimental model [47,48]. Selected brain active substances, such as corticotropin-releasing hormone (CRH), gonadotropin-releasing hormone (GnRH, luteinizing-hormone-releasing hormone, LHRH), thyrotropin-releasing hormone (TRH), galanin (GAL), neuropeptide Y (NPY), somatostatin (SOM), substance P (SP) and vasoactive intestinal polypeptide (VIP) were chosen for this study not only due to their wide distribution in the HPA, HPO and HPT axes, but also because of their specificities. Among other activities, these substances play a substantial role in numerous physiological processes; e.g., functioning as neurotransmitters and/or hormones to help maintain homeostasis. Moreover, previous studies reported that GnRH GAL, NPY, SOM and VIP have neuroprotective functions and are a promising target for therapy. However, dysregulation of the brain peptides in HPA, HPO, and HPT axes leads to homeostatic imbalance and may be implicated in the development of some chronic diseases or symptoms of depression, for example dysregulation of the HPA axis is correlated with depression in the animal model of PD [49] and HPA hyperactivity is linked to a heightened risk for depression and anxiety disorders in humans [50,51]. 

For this reason, the present study tested the hypothesis that a single subclinical dose of LPS from *S.* Enteritidis would induce dysregulation of various brain peptides such as CRH, GnRH, TRH, GAL, NPY, SOM, SP and VIP in selected clinically important brain sections and endocrine glands of the HPA, HPO and HPT axes. Therefore, the objectives of the present study were to determine the concentration of the key regulators of endocrine axes such as CRH, TRH and GnRH in the preoptic area (POA), the medial basal hypothalamus (MBH), the lateral hypothalamic area (LHA), the mammillary bodies (MB) and the stalk median eminence (SME). Moreover, the concentration of CRH was determined in the adrenal gland, the concentration of TRH in the thyroid gland and the concentration of GnRH in the ovaries. Additionally, this study was designed to detect the levels of GAL, NPY, SOM, SP, and VIP in: the selected sections of the hypothalamus and the pituitary gland such as POA, MBH, LHA, MB, SME, the adenohypophysis (AP), the neurohypophysis (NP) and the endocrine glands of the HPA, HPO, and HPT axes such as the adrenal gland, the thyroid gland, and the ovaries.

## 2. Results

Throughout the experiment, all the animals were evaluated daily by a veterinary surgeon (DVM, Ph.D.) as being clinically healthy and were free from any symptoms of disease. The pigs tolerated the treatment injection of the subclinical dose of LPS without distress or changes in appearance, rectal temperature or body weight. There was no difference found in health status between the LPS group and the control group. The differences in rectal temperature and body weight between the groups were not statistically significant. Each morning and afternoon measurement of the rectal temperature fell within the normal temperature range for both the LPS and the control pigs. The average rectal temperature during the experiment in the morning was determined as 39.02 ± 0.02 °C in the control group and 39.05 ± 0.03 °C in the LPS group, and in the afternoon as 39.02 ± 0.03 °C in the control group and 39.04 ± 0.03 °C in the LPS group, respectively. There were no statistical differences noted in the average daily weight gain between the control and the LPS groups. The average daily gain throughout the whole period of the experiment was determined as 272.0 ± 2.8 g in the control group and 268.9 ± 4.6 g in the LPS group, respectively. The other data of temperature and body weight has been included in our previous publication [52].

**Figure 1:**
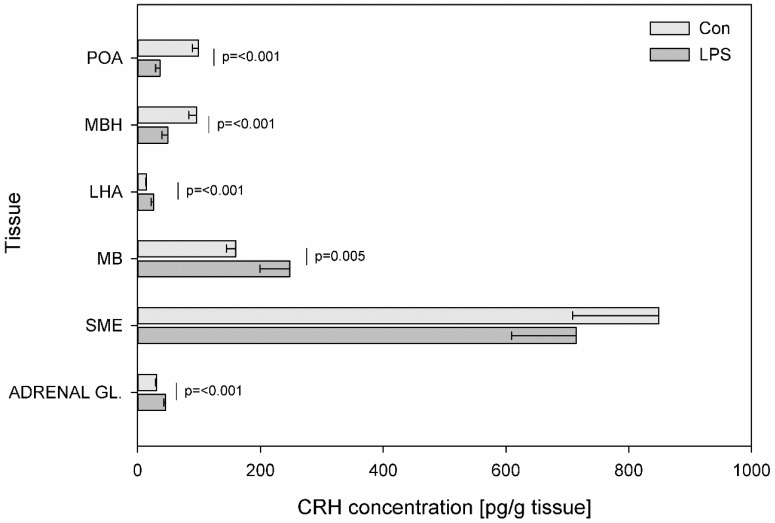
Concentration of corticotropin-releasing hormone in the selected brain regions and in the adrenal gland. Concentration of corticotropin-releasing hormone (CRH, pg/g of tissue) in the different brain regions: the preoptic area (POA), the medial basal hypothalamus (MBH), the lateral hypothalamic area (LHA), the mammillary bodies (MB), the stalk median eminence (SME) and in the adrenal gland (ADRENAL GL.) in the control group (Con, n = 5) and in the group after LPS exposure (LPS, n = 5). Dysregulation of CRH levels was observed in the LPS group seven days after a single subclinical dose of LPS from *S.* Enteritidis. Mean ± SD. Values were statistically compared using Student’s *t*-test (*p* < 0.05).

Figure 1 presents the mean levels of CRH in the control and LPS groups. LPS administration effected a decrease in CRH levels in MBH (from 95.82 ± 12.62 to 49.04 ± 9.24 pg/g tissue) (*p* ≤ 0.001) and in POA (from 98.92 ± 10.13 to 36.25 ± 7.04 pg/g tissue) (*p* ≤ 0.001) and an increase in the CRH levels in LHA (from 14.08 ± 1.18 to 26.14 ± 4.38 pg/g tissue) (*p* ≤ 0.001), in MB (from 159.88 ± 15.48 to 247.43 ± 48.36 pg/g tissue) (*p* = 0.005) and in the adrenal gland (from 30.70 ± 1.39 to 45.34 ± 2.55 pg/g tissue) (*p* ≤ 0.001).

**Figure 2:**
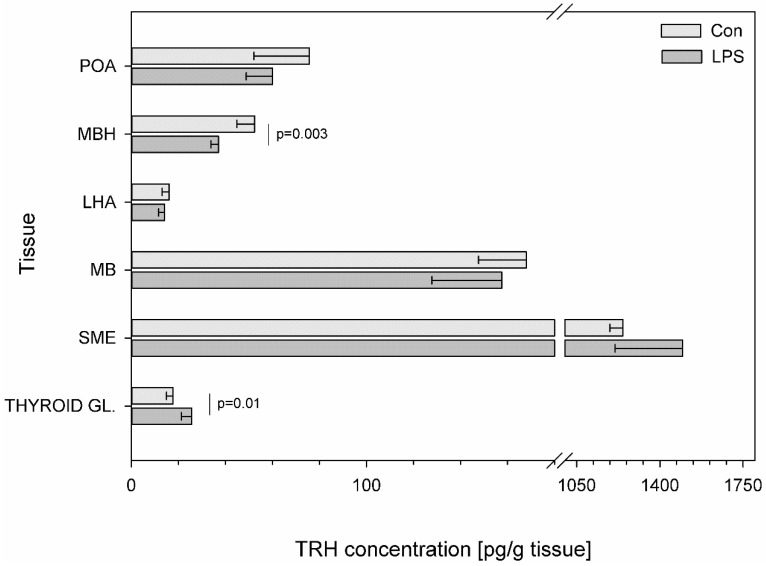
Concentration of thyrotropin-releasing hormone in the selected brain regions and in the thyroid gland. Concentration of thyrotropin-releasing hormone (TRH, pg/g of tissue) in the different brain regions: the preoptic area (POA), the medial basal hypothalamus (MBH), the lateral hypothalamic area (LHA), the mammillary bodies (MB), the stalk median eminence (SME) and in the thyroid gland (THYROID GL.) in the control group (Con, n = 5) and in the group after LPS exposure (LPS, n = 5). Dysregulation of CRH levels was observed in the LPS group seven days after a single subclinical dose of LPS from *S.* Enteritidis. Mean ± SD. Values were statistically compared using Student’s *t*-test (*p* < 0.05).

Subclinical LPS significantly changed the concentration of TRH in only two examined structures. After LPS administration, an increase in TRH concentration was observed in the thyroid gland (from 17.77 ± 2.85 to 25.64 ± 4.38 pg/g tissue) (*p* = 0.01) while within the MBH, a decrease in TRH levels was noted (from 52.43 ± 7.53 to 36.99 ± 3.05 pg/g tissue) (*p* = 0.003) (Figure 2).

**Figure 3:**
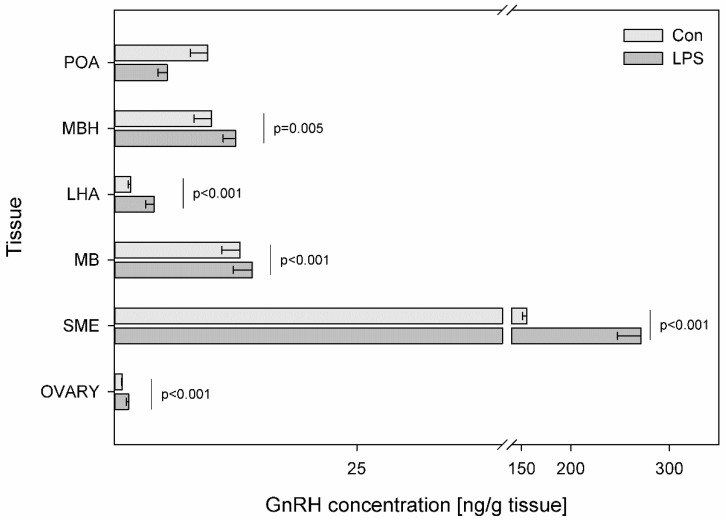
Concentration of gonadotropin-releasing hormone in the selected brain regions and in the ovary. Concentration of gonadotropin-releasing hormone (GnRH, ng/g of tissue) in the different brain regions: the preoptic area (POA), the medial basal hypothalamus (MBH), the lateral hypothalamic area (LHA), the mammillary bodies (MB), the stalk median eminence (SME) and in the ovary (OVARY) in the control group (Con, n = 5) and in the group after LPS exposure (LPS, n = 5). Dysregulation of CRH levels was observed in the LPS group seven days after a single subclinical dose of LPS from *S.* Enteritidis. Mean ± SD. Values were statistically compared using Student’s *t*-test (*p* < 0.05).

Differences in GnRH levels between LPS and control groups in all studied tissues were found to be statistically significant, except for POA. LPS administration produced an over two-fold increase in GnRH content in LHA (from 1.70 ± 0.25 to 4.09 ± 0.85 ng/g tissue) (*p* < 0.001) and an almost two-fold increase in SME (from 155.14 ± 4.3 to 271.47 ± 24.0 ng/g tissue) (*p* < 0.001) and in ovaries (from 0.82 ± 0.07 to 1.46 ± 0.23 ng/g tissue) (*p* < 0.001). Compared to the control, the values of GnRH also increased substantially following the LPS injection from 10.01 ± 1.80 to 12.50 ± 1.28 (*p* = 0.005) in MBH and from 12.96 ± 1.89 to 14.22 ± 1.96 in MB (*p* ≤ 0.001) (Figure 3). 

**Figure 4:**
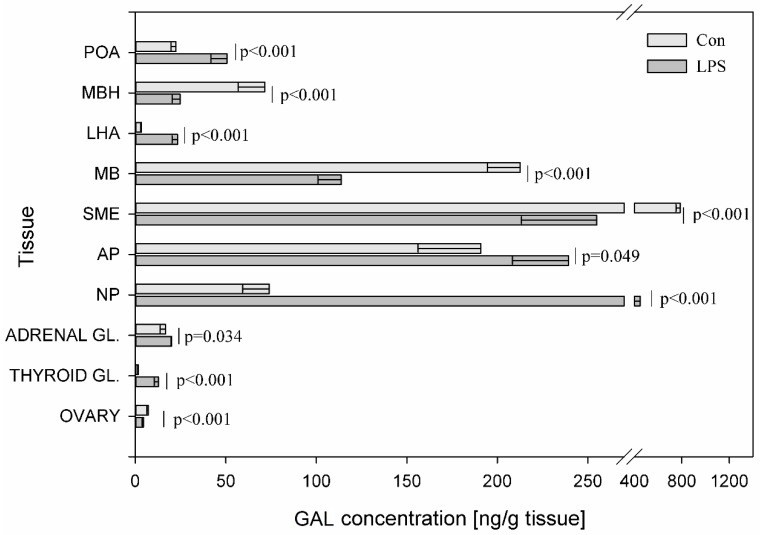
Concentration of galanin in the selected brain regions and the endocrine glands of HPA, HPT, and HPO axes. The concentration of galanin (GAL, ng/g of tissue) in different brain regions: preoptic area (POA), medial basal hypothalamus (MBH), lateral hypothalamic area (LHA), mammillary bodies (MB), stalk median eminence (SME), adenohypophysis (AP), neurohypophysis (NP) and in the endocrine glands, such as the adrenal gland (ADRENAL GL.), the thyroid gland (THYROID GL.) and the ovary (OVARY) in the control group (Con, n = 5) and in the group after LPS exposure (LPS, n = 5). Dysregulation of GAL levels was observed in the LPS group seven days after a single subclinical dose of LPS from *S.* Enteritidis. Mean ± SD. The values were statistically compared using Student’s *t*-test (*p* < 0.05).

Among all studied brain peptides, statistically significant changes in the concentration of GAL after LPS administration was observed in all examined structures. LPS administration caused a statistically significant decrease at *p* < 0.001 in the content of GAL in the SME (from 786.53 ± 36.17 to 254.83 ± 41.41 ng/g tissue), in the MBH (from 71.47 ± 14.56 to 24.88 ± 4.45 ng/g tissue), in the MB (from 212.65 ± 18.12 to 113.71 ± 12.87 ng/g tissue), in the ovaries (from 7.23 ± 0.88 to 4.57 ± 0.75 ng/g tissue) and an increase in the other studied structures. The most visible changes after subclinical LPS administration were observed in LHA, where GAL exceeded by almost seven-fold its concentration and in the thyroid, where GAL exceeded its concentration by above seven-fold. LPS induced an increase in GAL levels in the POA from 22.43 ± 2.67 to 50.62 ± 8.81 ng/g tissue (*p* < 0.001), in the LHA from 3.43 ± 0.56 to 23.38 ± 3.05 ng/g tissue (*p* < 0.001), in the NP from 74.00 ± 14.67 to 449.21 ± 84.38 ng/g tissue (*p* < 0.001), in the AP from 190.95 ± 34.73 to 239.30 ± 30.93 (*p* = 0.049) in the adrenal gland from 16.74 ± 2.95 to 20.14 ± 0.49 (*p* < 0.034) and in the thyroid gland from 1.73 ± 0.37 to 12.84 ± 2.31 ng/g tissue (*p* < 0.001) (Figure 4).

**Figure 5:**
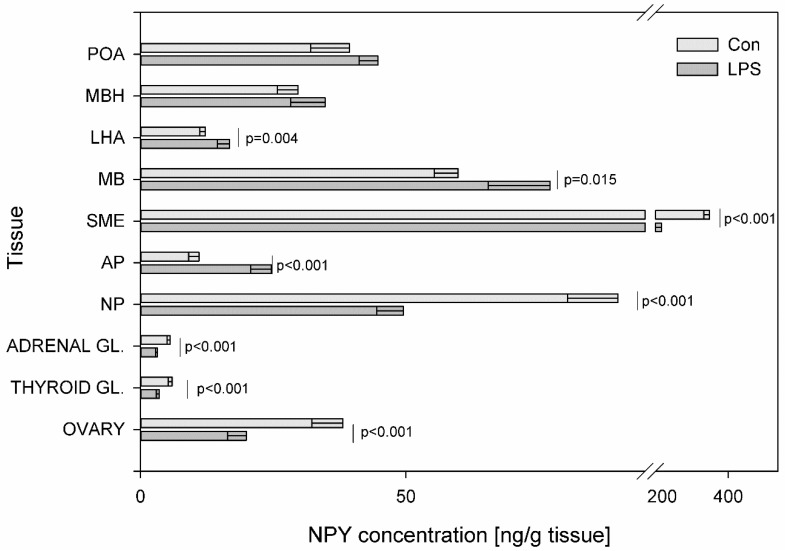
Concentration of neuropeptide Y in the selected brain regions and the endocrine glands of HPA, HPT, and HPO axes. The concentration of neuropeptide Y (NPY, ng/g of tissue) in different brain regions: preoptic area (POA), medial basal hypothalamus (MBH), lateral hypothalamic area (LHA), mammillary bodies (MB), stalk median eminence (SME), adenohypophysis (AP), neurohypophysis (NP) and in the endocrine glands, such as the adrenal gland (ADRENAL GL.), the thyroid gland (THYROID GL.) and the ovary (OVARY) in the control group (Con, n = 5) and in the group after LPS exposure (LPS, n = 5). Dysregulation of GAL levels was observed in the LPS group seven days after a single subclinical dose of LPS from *S.* Enteritidis. Mean ± SD. The values were statistically compared using Student’s *t*-test (*p* < 0.05).

A statistically significant decrease at *p* < 0.001 in NPY levels was noted in SME (from 343.08 ± 17.54 to 197.44 ± 27.32 ng/g tissue), NP (from 89.90 ± 9.46 to 49.49 ± 5.01 ng/g tissue) and in all endocrine glands of HPA, HPO and HPT axes. In particular, the decrease in the NPY concentration after LPS administration was observed within the adrenal gland (from 5.58 ± 0.57 to 3.18 ± 0.35 ng/g tissue), in the thyroid gland (from 5.97 ± 0.76 to 3.56 ± 0.55 ng/g tissue) and in the ovary (from 38.08 ± 5.84 to 19.93 ± 3.56 ng/g tissue). Compared to the control, the values of NPY increased significantly following the LPS injection from 11.01 ± 1.93 to 24.62 ± 3.89 ng/g tissue (*p* < 0.001) in the AP, from 12.20 ± 1.06 to 16.76 ± 2.28 ng/g tissue (*p* = 0.004) in the LHA and from 59.82 ± 4.46 to 77.12 ± 11.65 (*p* = 0.015) ng/g tissue in the MB (Figure 5).

**Figure 6:**
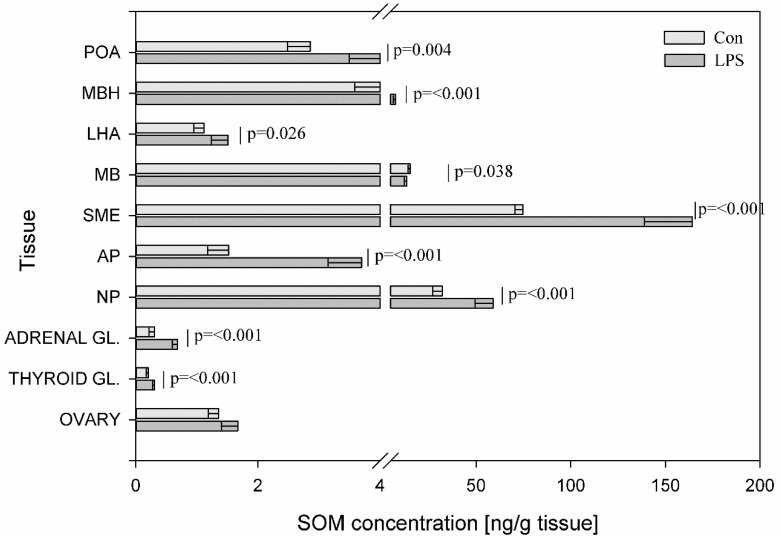
Concentration of somatostatin in the selected brain regions and the endocrine glands of HPA, HPT, and HPO axes. The concentration of somatostatin (SOM, ng/g of tissue) in different brain regions: preoptic area (POA), medial basal hypothalamus (MBH), lateral hypothalamic area (LHA), mammillary bodies (MB), stalk median eminence (SME), adenohypophysis (AP), neurohypophysis (NP) and in the endocrine glands, such as the adrenal gland (ADRENAL GL.), the thyroid gland (THYROID GL.) and the ovary (OVARY) in the control group (Con, n = 5) and in the group after LPS exposure (LPS, n = 5). Dysregulation of GAL levels was observed in the LPS group seven days after a single subclinical dose of LPS from *S.* Enteritidis. Mean ± SD. The values were statistically compared using Student’s *t*-test (*p* < 0.05).

SOM levels underwent LPS-induced changes in most of the studied tissues. The administration of LPS caused changes in SOM levels in nine out of ten investigated tissues. Its concentration increased within the POA, MBH, LHA, SME, AP, NP adrenal and thyroid glands and decreased in MB. A statistically significant decrease at *p* = 0.038 in SOM levels in MB was noted from 15.02 ± 0.93 to 13.26 ± 1.29 ng/g tissue. Compared to the control, the values of SOM increased substantially following the injection of LPS from 4.10 ± 0.51 to 7.36 ± 1.21 ng/g tissue (*p* < 0.001) in the MBH, from 2.86 ± 0.37 to 4.08 ± 0.59 ng/g tissue (*p* = 0.004) in the POA, from 1.12 ± 0.17 to 1.51 ± 0.27 ng/g (*p* = 0.026) in the LHA and from 32.13 ± 5.09 to 58.91 ± 9.33 (*p* < 0.001) ng/g tissue in the NP (Figure 6).

**Figure 7:**
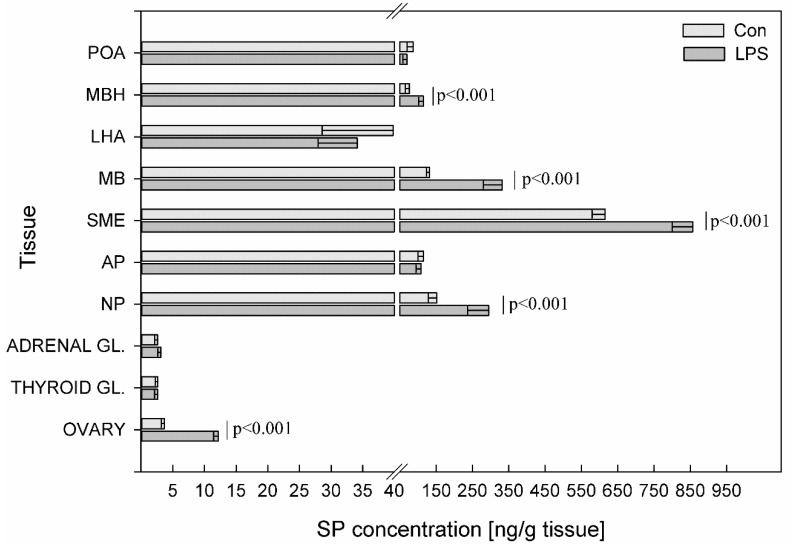
Concentration of substance P in the selected brain regions and the endocrine glands of HPA, HPT, and HPO axes. The concentration of substance P (SP, ng/g of tissue) in different brain regions: preoptic area (POA), medial basal hypothalamus (MBH), lateral hypothalamic area (LHA), mammillary bodies (MB), stalk median eminence (SME), adenohypophysis (AP), neurohypophysis (NP) and in the endocrine glands, such as the adrenal gland (ADRENAL GL.), the thyroid gland (THYROID GL.) and the ovary (OVARY) in the control group (Con, n = 5) and in the group after LPS exposure (LPS, n = 5). Dysregulation of GAL levels was observed in the LPS group seven days after a single subclinical dose of LPS from *S.* Enteritidis. Mean ± SD. The values were statistically compared using Student’s *t*-test (*p* < 0.05).

LPS administration resulted in a statistically significant increase at *p* < 0.001 of SP levels in MBH (from 76.13 ± 10.77 to 115.12 ± 12.75 ng/g tissue), MB (from 132.31 ± 9.30 to 332.20 ± 52.40 ng/g tissue), SME (from 615.63 ± 36.12 to 856.74 ± 56.21 ng/g tissue), NP (from 151.5 ± 23.00 to 294.94 ± 57.69 ng/g tissue) and the ovaries (from 3.69 ± 0.52 to 12.17 ± 0.71 ng/g tissue). In the other studied structures, no significant statistical changes in the concentration of SP were observed between the LPS group and the control group (Figure 7).

**Figure 8:**
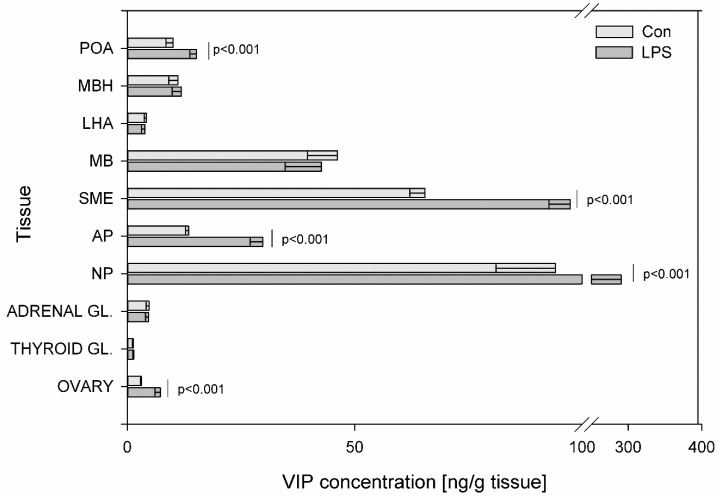
Concentration of vasoactive intestinal polypeptide in the selected brain regions and the endocrine glands of HPA, HPT, and HPO axes. The concentration of vasoactive intestinal polypeptide (VIP, ng/g of tissue) in different brain regions: preoptic area (POA), medial basal hypothalamus (MBH), lateral hypothalamic area (LHA), mammillary bodies (MB), stalk median eminence (SME), adenohypophysis (AP), neurohypophysis (NP) and in the endocrine glands, such as the adrenal gland (ADRENAL GL.), the thyroid gland (THYROID GL.) and the ovary (OVARY) in the control group (Con, n = 5) and in the group after LPS exposure (LPS, n = 5). Dysregulation of GAL levels was observed in the LPS group seven days after a single subclinical dose of LPS from *S.* Enteritidis. Mean ± SD. The values were statistically compared using Student’s *t*-test (*p* < 0.05).

The highest concentration of all determined peptides observed during the present study in control animals occurred in SME (Figure 1, Figure 2, Figure 3, Figure 4, Figure 5, Figure 6 and Figure 7), except VIP, whose highest concentration was observed in NP. Subclinical LPS induced a statistically significant increase at *p* < 0.001 of VIP levels in POA (from 10.05 ± 1.58 to 15.18 ± 1.48 ng/g tissue), SME (from 65.45 ± 3.31 to 97.41 ± 4.69 ng/g tissue), AP (from 13.47 ± 0.72 to 29.80 ± 2.81 ng/g tissue), NP (from 94.21 ± 13.11 to 290.45 ± 48.13 ng/g tissue) and in ovaries (from 3.07 ± 0.18 to 7.24 ± 1.18 ng/g tissue) (Figure 8).

## 3. Discussion

This study is the first one to demonstrate that a low single dose of LPS *S.* Enteritidis, which does not result in any clinical symptoms of disease (subclinical LPS), can dysregulate levels of CRH, GnRH, TRH, GAL, NPY, SOM, SP, and VIP in selected clinically important brain sections and endocrine glands of HPA, HPO, and HPT axes. The principal findings of exposure to subclinical LPS are in agreement with previous studies. Previously, our studies showed that a single low dose of LPS *S.* Enteritidis modulated the main porcine enteric neuropeptides in intestines and changed the number and chemical coding of intramural nerves within the porcine gallbladder wall [53,54,55]. Moreover, subclinical LPS from *S.* Enteritidis increased levels of dopamine in the brain and neuropeptides in the cervical lymph nodes seven days after LPS administration [52]. In addition, Lew et al. [56] concluded that recurrent exposure to subclinical LPS *E. coli* O55 induced cardiac fibrosis in mice. The results obtained in the study clearly show that even a low dose of LPS *S.* Enteritidis, which does not result in any clinical symptoms of disease, can affect the chemistry of brain structure and dysregulate selected bioactive substance from brain sections and endocrine glands. Dysregulation of brain peptides in structures of the endocrine axes can be a feature of homeostasis disruption and may contribute to various dysfunctions. It should be noted that dysregulation of selected peptides in hormonal axes under the influence of subclinical LPS *S.* Enteritidis indicates that it is not ethical in the scientific studies to expose humans to even such low doses of LPS with unknown long-term consequences. Apart from using LPS to develop drugs ranging from cancer therapy and vaccines to immunostimulants, LPS in subclinical doses has been injected into normal human volunteers in several studies which were mostly concentrated only on blood parameters because of the inaccessibility of major tissues for biopsy [57,58]. Our findings draw attention to the importance of the aspects of unknown long-term consequences of the influence of subclinical LPS from *S.* Enteritidis on the body, which should be analyzed both in the research strategies and the pharmaceutical industry.

It should be noted that our current study focuses on peptide levels extracted using solid phase extraction procedures from both neural and non-neuronal cells in studied tissues from the brain and selected organs. The different types of non-neuronal cells in particular glia, epithelial cells, pericytes and endothelial cells in the brain and various types of cells in peripheral axis organs display a wide heterogeneity within each non-neuronal cell type. Since there are no tools currently available to sufficiently distinguish peptide secretion or storage between these subpopulations [59], the indication of peptide levels from all types of neuronal and non-neuronal cells seems the most rational in the comparison between studied LPS and control groups. Moreover, to better understand the function of the key regulators of endocrine axes such as CRH, TRH and GnRH in homeostasis and pathologies it is necessary to elucidate the participation of active substances such as GAL, NPY, SOM, SP and VIP in the activity of HPA, HPT and HPO axes. 

There are many pathophysiological conditions that are associated with HPA axis dysregulation, such as aging, affective disorders and metabolic diseases. Dysregulation of HPA during the neonatal development period may lead to increased risk of neurological, cardiovascular, and metabolic diseases in later life [60]. CRH is the brain peptide triggering the response to stress and the mediator/stimulator of HPA axis. Our data demonstrated that subclinical LPS effected dysregulation of CRH levels in all examined tissues except SME. The concentration of CRH decreased in POA, MBH and increased in LHA, MB and adrenal gland seven days after LPS administration (Figure 6). This CRH dysregulation can be connected with other studied peptides levels. The production and secretion of CRH in the hypothalamus are mainly regulated by a negative feedback mechanism, but some active substances such as SOM also have an effect on these processes. Reports have indicated that the activation of brain SOM signaling in different brain sites exerts an anti-stress action and alleviates many components of the stress response involving brain CRH signaling [61]. We hypothesize that the significant increases of SOM levels in the studied brain structures seven days after LPS administration (Figure 6) are connected with an attempt to regulate CRH concentration. A similar hypothesis can be considered in the adrenal gland where subclinical LPS induced the increase of SOM concentration (Figure 6). In a previous study, the response to a stress factor such as LPS during experimental endotoxemia, VIP and GAL mRNA levels increased in the adrenal gland to affect steroid production by adrenocortical cells [62]. Similarly, in the current study, the level of adrenal GAL significantly increased during subclinical LPS treatment (Figure 4). However, in contrast, the level of adrenal VIP did not change significantly in LPS group as compared with the control (Figure 8). The observed changes of adrenal SOM and GAL in the current study are probably connected with an attempt to maintain homeostasis, but it is difficult to explain these changes in a very clear way. The effects of adrenal GAL on the adrenal secretion are still controversial and the anti-stress properties of adrenal SOM require further examination. 

Besides the correlation of the HPA axis with stress, there is the integration of the HPA axis and the regulation of feeding. Dysregulation of the HPA axis contributes to obesity and the development of insulin resistance [63,64]. Acute activation of the HPA axis exerts anorexigenic-increased CRH levels and an additional decrease in NPY secretion. The prolonged activation of the HPA axis with the attenuation of negative feedback in the HPA axis, which inhibits CRH and stimulates NPY expression, can be associated with abdominal obesity [65]. Recent evidence in rodents and humans suggests that systemic, low levels of LPS from the gut bacteria play an important role in obesity [66]. There is a close relationship between obesity and the pathogenesis of diabetes mellitus, hypertension, and atherosclerosis. Long et al. [67] demonstrated the establishment of insulin resistance in rat, following long-term constitutive over-expression of NPY in the hypothalamus. Sergeyev et al. [68] suggested that four hours after an injection of a single high dose of LPS *Salmonella* Abortusequi induced signal-mediating factors such as a decrease in mRNAs encoding GAL synthesis in LHA. A chronic low dose LPS treatment increased body weight gain and energy intake but had no effect on inflammatory mediators [69]. In the current study, pigs tolerated the injection of a single subclinical dose of LPS from *S.* Enteritidis without statistically significant differences in body weight between the control animals and the LPS group during all periods of the experiment. Nevertheless, dysregulation of several peptides involved in the regulation of feeding was observed (Figure 1, Figure 4, Figure 5 and Figure 6). Some of the peptides involved in the regulation of feeding (such as NPY, GAL) stimulate eating, others (like CRH and SOM) inhibit appetite. In the LHA—the feeding center of the hypothalamus, an increase was observed in both stimulators such as NPY, GAL and appetite inhibitors such as CRH and SOM in the LPS group compared to the control. It seems likely that the high content of the mentioned peptides in LHA seven days after LPS administration could participate in the maintenance of the body weight gain at the same level as in the control group (Figure 1, Figure 4, Figure 5 and Figure 6).

The HPT axis has an important role in the control of energy expenditure and body metabolism. Thus, the modulation of the HPT axis by peripheral signals of energy deposition is of great significance for the maintenance of metabolic homeostasis. Dysregulation of the HPT axis function occurs during a variety of illnesses. Suppression of the central part of the HPT axis constitutes one of the mechanisms of protection against excessive catabolism by maintenance of low serum thyroid hormone levels during illness. TRH, which is the key peptide of the HPT axis, apart from anti-aging effects has a fundamental role in the regulation of metabolic and hormonal functions [70]. The results of the present study demonstrated a decrease of TRH value in MBH and in the thyroid gland after subclinical LPS administration compared to saline-treated control pigs (Figure 2). In turn, while in the previous study acute illness was induced by a high dose of LPS from *E. coli* O127:B8, the expression of TRH mRNA did not change in hypothalamus compared with control mice one day after LPS administration [71]. The decrease in TRH level in MBH seven days after subclinical LPS administration may be connected with a decrease in GAL levels in MBH after LPS administration (Figure 1 and Figure 4), although regarding the regulation of TRH secretion, the role of GAL, if one exists, is still controversial [72]. The current data provide new evidence for the influence of subclinical LPS on TRH secretion in the thyroid gland. Our study demonstrated that an increase of TRH level in the thyroid gland after LPS administration (Figure 2) may be connected with the direct or indirect influence of subclinical LPS on the TRH level. It has already been well-established that cells of the thyroid gland express TRH mRNA as well as true TRH at protein levels and TRH probably inhibits the release of thyroid hormones as an antagonist to TSH [73,74]. Peptides like GAL, NPY, and SOM, which are also produced in the thyroid gland, may play a role in thyroid physiology and have an influence on TRH level [75]. The significant decrease in NPY level and the increase of GAL and SOM levels in the thyroid gland following subclinical LPS administration observed in the current study are probably connected with the significant increase of TRH in the LPS group compared to the control animals (Figure 2, Figure 4 and Figure 5). Although the role of GAL in the thyroid hormone secretion is still unknown, in recent years evidence has been accumulating in the literature that SOM and NPY could modify thyroid function. SOM seems to be involved as a local inhibitor of thyroid function. NPY has convincingly been demonstrated to enhance the inhibitory action of noradrenaline on TSH-induced thyroid hormone secretion and, similar to noradrenaline, inhibits thyroidal blood flow [75,76].

Reproductive maturation and function are associated with body metabolic status. The HPO axis is modulated by nutritional/energy status and gonadal function. Within this neuroendocrine axis, GnRH is considered to be the key element. The present study was performed on the juvenile gilts and indicated that the level of GnRH increased in MBH, LHA, MB, SME, and ovary seven days after subclinical LPS administration (Figure 3). The mechanism and the increase in pulsatile release of GnRH is essential for the onset of puberty [77]. GnRH secretion is mainly influenced by NPY and GAL. NPY and GAL play a role in modulating pulsatile GnRH release and the mechanism of the onset of puberty. NPY might be acting at the median eminence as a physiological stimulus for pre-ovulatory GnRH release [78]. NPY influences the binding of GnRH to its receptors in the rat anterior pituitary and the increase LH release from anterior pituitary cells in response to GnRH. We hypothesize that dysregulation of GAL levels in LPS group (Figure 4) may be due to GnRH levels. GAL is a potent regulator of GnRH neuron and GAL binding levels the increased in the POA across puberty in both male and female rats [77,79]. Like the hypothalamic effects of GAL on GnRH neurosecretion, the pituitary effects of the peptide are important for the occurrence of preovulatory LH surges in females. GnRH, NPY, and GAL play a role in the induction of the LH surges and gonadotrope sensitivity to the neuropeptide will also be enhanced under the steroid conditions which result in preovulatory LH surges. GAL alone does not directly stimulate LH secretion, but by modulation of GnRH, GAL inhibits GnRH-stimulated LH secretion [80]. The increase in GnRH levels and dysregulation of GAL levels in study juvenile gilts after subclinical LPS *S.* Enteritidis administration (Figure 3 and Figure 4) may hypothetically be associated not only with metabolism and reproduction but also with the neurodegeneration processes. Changes in HPO axis active substances are implicated in the neuropathology of AD [81]. 

The emerging evidence suggests that peptides dysregulations of HPA, HPT, and HPO axes are implicated in neurodegenerative diseases and neuropsychiatric disorders [51,81,82]. It is well- known that neurodegenerative processes and associated diseases are correlated with aging but some of them have an onset or are present in the younger population. Neurodegenerative processes can begin long before any symptoms become apparent and are still not well understood. Studied brain peptides can influence each other, but also can impact on different active substances such as dopamine. Previous research suggests that SP may be important in the early stages of PD pathogenesis to maintain brain dopamine (DA) levels [83]. Our previous study demonstrated DA increase in the prefrontal cortex and substantia nigra seven days after subclinical LPS administration [52]. Our current study shows a statistically significant increase of SP levels in MBH, MB, SME, and NP (Figure 7) Wang et al. [84] found that mice lacking endogenous SP were more resistant to LPS-induced nigral dopaminergic neurodegeneration. Zhu at al. [85] found that SP can trigger microglial activation and subsequent stimulation of the production of pro-inflammatory factors in the brain. SP has the ability to stimulate DA release. Injections of SP into the substantia nigra increased DA levels in the striatum and in the frontoparietal cortex in rats [86]. Since it has been suggested that striatal and cortical DA release is regulated by nigral SP, the increase of SP expression in the current study (Figure 7) may be due to a greater release from brain structures to maintain brain DA levels or it may be due to an unknown subclinical molecular response, which requires further studies. Because of the leading role of microglia in pathologies of neurodegenerative diseases, the study of microglia modulatory factors such as LPS and knowledge about microglia deactivating peptides has become very important [87]. Increasing evidence [88,89,90] suggests that NPY might have a neuroprotective function on LPS-induced microglial activation. GAL also has neurotrophic properties and has been directly implicated in neurodegenerative diseases. GAL upregulation in degenerative tissue may represent a neuroprotective mechanism in the AD and Lewy body disorders [91,92,93]. The inhibitory role of GAL in cognitive processes, taken together with the overexpression of GAL in AD, during neuronal development and after stimulation with estrogen, suggests that GAL has been implicated in neuroendocrine modulation and in the survival of neurons in regions undergoing neurodegeneration [94,95]. Another microglia-deactivating factor is VIP, which can inhibit LPS-stimulated production of pro-inflammatory mediators [96]. Song et al. [97] suggested a protective role for VIP in AD through the reduction amyloid beta and modulation of microglial function. The study performed in the rat model of PD generated by injecting LPS into the brain’s substantia nigra showed that SOM decreased the number of activated microglial cells and was also able to prevent neuronal cell death and reduce the production of inflammatory markers by the substantia nigra [98]. Considering the above information about the possible role of studied peptides in the prevention of neurodegenerative processes it should be pointed out that peptides such as CRH, TRH, GnRH, GAL, NPY, SOM and VIP have multiple functions in neoplasia and are linked to oncology research and treatment. Moreover, many tumors can overproduce GnRH, CRH, NPY, VIP, SOM etc. SOM and its analogues are highly effective in reducing hormonal symptoms caused by endocrine tumors. These SOM analogues form an integral part of the routine therapy of several such tumors [99]. Besides various uses of GnRH agonists and antagonists in gynacology, reproductive medicine and oncology, recent studies indicate that GnRH can be employed to guide anticancer and imaging agents directly to cancer cells, preventing normal cells from unnecessary exposure [100]. The human GnRH are important regulatory components in the regulation of cell proliferation in both hormone-dependent and independent types of tumors [101].

Taken together, the pathophysiological role and potential therapeutic applications of study peptides in connection with our obtained results indicate that there is general agreement that proteins and peptides control most of our vital functions. They can change their levels under pathological agents and physiological factors, such as the growth and development of the living organism. Changes in peptides levels can help maintain homeostasis or result directly in a disease. Dysregulation of peptides can be important for cancer, metabolic, neurodegenerative and neuropsychiatric disorders. In fact, not all dysregulations are always dangerous because the human body is biologically programmed to maintain internal homeostasis through negative feedback-regulatory mechanisms. However, maladaptive actions of an initially adaptive strategy in correlation with a factor such as LPS can potentially affect the consequence of chronic organic disease, because LPS and its markers, such as 3-hydroxy fatty acids can remain in tissues and organs for many years [46,102]. Additionally, it should be pointed out that knowledge of the distribution and degradation of LPS from different pathogens is limited because of problems with specificity and low endotoxin recovery in measuring this potent and multifunctional compound in body tissues [45,103]. There is a general agreement that LPS can be very dangerous with its inflammatory symptoms leading to septic shock. LPS plays a very powerful role in the development of carcinogenesis, neurodegeneration and in metabolic disturbances. On the other hand, the previous study suggested that low dose LPS from *S.* Enteritidis preconditioning prevented subsequent LPS-induced severe liver damage [104]. Furthermore, administration of LPS from *S.* Enteritidis and LPS from Pantoea agglomerates had antitumor effects and reduced tumor growth in mice and humans [105]. Therefore, considerations of LPS variability from bacterial sources and the long-term consequences of different doses of LPS must be taken into account. Due to the fact that even a single low dose of LPS *S.* Enteritidis which does not cause any clinical symptoms of diseases may affect the brain peptides, a reasonable research strategy is required. It seems very important to look for changes in detectable LPS *Salmonella* spp. in chronic diseases, especially in patients with symptomatic and asymptomatic *Salmonella* infections in the past. The prediction is that the elimination of LPS *Salmonella* should be of value in the treatment of chronic non-communicable diseases as the curative treatment of *H. pylori* significantly reduced cancer incidence with *H. pylori* [106]. Defining the factors associated with the pathological process much earlier before the damage of compensatory mechanisms can prevent diseases.

## 4. Conclusions

In summary, the obtained results clearly show that subclinical LPS *S.* Enteritidis can affect the chemistry of brain structure and dysregulate bioactive substance from selected brain sections and endocrine glands. We proved that even a low single dose of LPS *S.* Enteritidis, which does not result in any clinical symptoms of disease, can change the concentration of brain peptides such as CRH, GnRH, TRH, GAL, NPY, SOM, SP, and VIP in selected brain sections of the hypothalamus, pituitary gland and in endocrine glands of HPA, HPO, and HPT axes, which may induce their dysregulation. The exact mechanisms by which LPS can affect major neuroendocrine axes are not fully understood and require further studies. Since dysregulation of brain peptides in the mentioned structures may be a feature of homeostasis disruption and may contribute to various dysfunctions, it should be pointed out that considerations of different LPS biological activity depending on various bacterial sources and the long-term consequences of LPS in different doses must be taken into account in the research strategies and the pharmaceutical industry.

## 5. Materials and Methods

### 5.1. Animal Housing and LPS Administration

The study was conducted on ten juvenile crossbred female pigs (Pietrain × Duroc). The experiment was carried out when the pigs were 8–9 weeks of age, with their body weights of 16–18 kg. The animals were maintained for 2 weeks prior to the beginning of the experiment so as to allow adaptation to the new environment. The pigs were kept under standard laboratory conditions and fed commercial feed suitable for pigs of this age group. The animals were assessed by a veterinary surgeon (DVM, Ph.D.); clinically healthy pigs which tested negative for *Salmonella* spp. in fecal samples were further qualified for the experiment.

After the adaptation period of 2 weeks, pigs included in the experiment were randomly assigned to two groups (five pigs in each): a control (Con) and an experimental (LPS) group. Animals from both groups received premedication, in accordance with the pattern previously described by Mikołajczyk [107] with intramuscular injection of atropine (Atropinum Sulfuricum Polfa Warszawa S.A., Warsaw, Poland, 0.035 mg/kg b.w.), ketamine (Bioketan, Vetoquinol-Biowet Sp. z o.o., Gorzów Wielkopolski, Poland, Lure, France, 7.0 mg/kg b.w.) and medetomidine (Cepetor, CP-Pharma Handelsges mbH, Burgdorf, Germany, 0.063 mg/kg b.w.). The premedication of animals allowed accuracy and safety (for the investigators) of LPS injections. Under premedication, the control animals were injected with a 10 mL of 0.9% NaCl (sodium chloride 0.9% WET Baxter, 9g/1000mL, Baxter Sp. z o.o., Warsaw, Poland) saline solution into the marginal ear vein, while pigs of the experimental group received in the same way (i.e., intravenously) LPS from *Salmonella enterica* serotype Enteritidis (catalogue no. L7770 Sigma, Aldrich, Taufkirchen, Germany) at a dose of 5 μg/kg b.w. (in 10 mL saline solution). Such a dose has been previously referred to as a “low single”, “subclinical” dose which does not result in any clinical symptoms of disease [52,53]. All procedures and drugs were managed and administered by a veterinary surgeon (DVM, Ph.D.), who was responsible for conducting a daily physical examination of each animal during the experiment. The observations made by the animal care staff as well as the measurements of temperature and body weight, both in the control and LPS group, were taken into consideration by the veterinary surgeon in the clinical assessment of the pigs’ health status. The body weights were determined once a day in the morning. The rectal temperatures were measured using an animal digital thermometer in the morning and in the afternoon as described by Mikołajczyk and Złotkowska [52]. The body weight per day and rectal temperatures taken twice a day were presented as the mean from the group ± SD.

### 5.2. Tissue Collection

After a 7-day period, described as sufficient for the appearance of changes in the nervous system in the previous studies [52,53,54,55,108,109], all animals were pre-medicated (in the above-mentioned manner) and subjected to general anaesthesia using propofol (Scanofol, NORBROOK, Newry, Northern Ireland, IRL.PN, 4.5 mg/kg b.w. given intravenously) and then euthanized with pentobarbital (Morbital-mix of pentobarbital sodium 133.3 mg/mL with pentobarbital 26,7 mg/mL, Biowet-Puławy Sp. z o.o, Puławy, Poland, 60–70 mg/kg b.w., given intravenously).

Immediately after euthanasia, the adrenal gland, ovaries, and the thyroid gland were excised. Next, the brain was rapidly removed from the skull. The SME was easily detached from the MBH without being cut and was cut away at its junction with the pituitary gland and then AP and NP were isolated [110,111]. Next, the POA, the LHA, MBH and MB were dissected [112]. All sections were identified based on pig brain atlases [113,114]. All tissues were frozen in liquid nitrogen immediately after collection and were stored at −80 °C until processing for further analysis.

### 5.3. Brain Peptide Extraction from the Tissues

Selected peptides, such as CRH, TRH, GnRH, GAL, NPY, SOM, SP, and VIP were extracted from MBH, POA, LHA, SME, MB, AP, NP, ovaries, adrenal and thyroid glands in a two-step procedure described below.

#### 5.3.1. Sample Preparation and High-temperature Extraction

Brain peptide extracts from tissue were prepared in accordance with the Conlon procedure [115]. Briefly, frozen tissue was cut into small pieces, then 10 mL of hot 1 M acetic acid was added per gram tissue and boiled for 5 min. The samples were subsequently homogenized using Ultra Turax IKA T-25 (Jankel & Kunkel IKA, Germany) at RT for 5 min and centrifuged at 4 °C for 40 min at 4500× *g* (Eppendorf 5804). The supernatant was subject to a Solid-phase Extraction (SPE) step. 

#### 5.3.2. Solid-phase Extraction and Concentration 

The supernatants were filtered through syringe filters without pre-filtering (Millex-HV Filter, 0.45 µm, PVDF, Millipore, Burlington, MA, USA) or syringe filters with a graduated glass fiber pre-filter (Millex-HPF HV Filter, 0.45 μm, PVDF, Millipore). In order to obtain a final concentration of 0.1% (vol/vol) TFA was added to filtrates. Depending on the type and size of the sample, Spin columns (8 mg of C18 resin per spin column, Pierce, Rockford, IL, USA), or Sep-Pak Plus Light Cartridge (130 mg of C18 sorbent per cartridge, Waters, Milford, MA, USA), or Sep-Pak C18 Plus Short Cartridge (360 mg of C18 sorbent per cartridge, Waters, Milford, MA, USA, were used according to the manufacturer’s protocol using a Baker Vacuum Manifold SPE-12G unit (J.T. Baker, Gross Gerau, Germany). Samples underwent concentration on a miVac centrifugal vacuum concentrator, model DNA-23050-800 with SpeedTrap (Genevac Limited, UK) for 2 h and then an ALPHA 1–4 LSC freeze dryer (MARTIN CHRIST Gefriertrocknungsanlagen GmbH, Osterode am Harz, Germany) was used for lyophilization. The lyophilized samples were kept at −80 °C until analysis.

All the chemicals used for this investigation were of commercial origin with HPLC grade purity: glacial acetic acid (cat. no. 951503, J.T. Baker), trifluoroacetic acid—TFA (cat. no. 9470, J.T. Baker) and acetonitrile—LC-MS reagent (cat. no. 9821.1000, J.T. Baker).

### 5.4. Enzyme-Linked Immunosorbent Assay for Quantitative Determination of Selected Peptides (CRH, TRH, GnRH, GAL, NPY, SOM, SP, VIP) in Tissue Extracts 

CRH (cat. no. MBS267253) and TRH (cat. no. MBS265712) ELISA kits were purchased from MyBioSource. The detection range of the tests was 15.6-1000 pg/mL; 0.156–10 ng/mL respectively for each of them. The protocol provided by the manufacturer was followed. First, 100 μL of sample was placed on the plate and incubated for 90 min at 37 °C. The plate was then washed four times with a 350 μL of assay buffer and 100 μL of biotinylated Porcine CRH or biotinylated Porcine CRH was added and incubated at 37 °C for 60 min. After another washing cycle, enzyme-conjugate was added. After a 30 min incubation and a washing step, 100 μL of color substrate A was added to each well and the plates were incubated again for 1 h at RT. The reaction was terminated with a 100 μL/well of color substrate B. Absorbance was read at λ = 450 nm on Infinite 200 (Tecan) for each sample.

GnRH (cat. no. EK-040-02CE), NPY (cat. no. EK-049-03CE), SOM (cat. no. EK-060-14CE) and VIP (cat. no. EK-064-16CE) were bought from Phoenix Pharmaceuticals, Inc. The detection range of tests was 0–25 ng/mL, 0–100 ng/mL, 0–25 ng/mL and 0–25 ng/mL, respectively, for each of them. We used the protocol proposed by the manufacturer. Briefly, 50 μL of sample together with 25 μL of primary antibody and 25 μL of biotinylated peptide were placed on a plate and incubated for 2 h at room temperature. The plate was then washed four times with a 350 μL of assay buffer and 100 μL of streptavidin conjugated with HRP was then added. After the incubation lasting 1 h and a washing step, 100 μL of TMB substrate was added to each well and the plates were incubated again for 1 h at RT. The reaction was terminated with 100 μL/well of 2 N HCl. Absorbance was read at λ = 450 nm on Infinite 200 (Tecan) for each sample.

SP (cat. no. S-1180) and GAL (cat. no. S-1210) ELISA kits were purchased from Peninsula Laboratories International, Inc. Their detection ranges were 0–5 ng/mL and 0–10 ng/mL, respectively, for SP and GAL. We used the protocol proposed by the manufacturer. Briefly, 50 μL of standard or sample, together with 25 μL of the primary antibody, were incubated overnight at 4 °C. The plates were washed five times with EIA buffer and 100 μL of streptavidin conjugated with HRP were added. After 1 h incubation and a washing step, 100 μL of TMB substrate was added to each well and the plates were incubated for about 30–60 min at RT. The reaction was terminated with a 100 μL/well of 2 N HCl. Absorbance was read at λ = 450 nm on an Infinite 200 (Tecan) for each sample.

A four parameter ELISA curve was prepared for each determined neuropeptide (an Excel sheet was provided by Peninsula Laboratories service). Each sample was tested in duplicate and the peptide concentration was read from the curve. Peptide concentration was presented as the mean from group ± the standard deviation (SD) per g of tissue. 

### 5.5. Statistical Analysis

The data were reported as the mean values ± SD. The data distributions in the groups were examined with the theoretical normal distribution using Shapiro-Wilk’s test. The differences between the groups were analyzed with Student’s *t*-test. The results were considered statistically significant at *p* < 0.05. The calculations were done with SigmaPlot® 12 (Systat Software Inc., Cracow, Poland).

### 5.6. Ethics Statement

All experimental procedures received the approval of the Local Ethical Committee for Animal Experimentation in Olsztyn (decision no. 73/2015 from 29th Sept 2015). The Local Ethical Committee for Animal Experimentation in Olsztyn is located at the University of Warmia and Mazury in Poland and is affiliated to the National Ethics Commission for Animal Experimentation (Polish Ministry of Science and Higher Education in Warsaw). All animals included in the experiment were kept and treated in accordance with all institutional and national guidelines applicable within the Republic of Poland, as per the Federal Law of 15 January 2015 on Animal Welfare for Science and Education (Dz.U.2015.0.266).
